# Sun Exposure and Psychotic Experiences

**DOI:** 10.3389/fpsyt.2017.00107

**Published:** 2017-06-19

**Authors:** Izabela Pilecka, Sven Sandin, Abraham Reichenberg, Robert K. R. Scragg, Anthony David, Elisabete Weiderpass

**Affiliations:** ^1^Department of Psychosis Studies, Institute of Psychiatry, Psychology and Neuroscience, King’s College London, London, United Kingdom; ^2^Department of Medical Epidemiology and Biostatistics, Karolinska Institutet, Stockholm, Sweden; ^3^Department of Psychiatry, Icahn School of Medicine at Mount Sinai, Friedman Brain Institute, New York, NY, United States; ^4^Department of Psychiatry, Icahn School of Medicine at Mount Sinai, Mindich Institute of Child Development, New York, NY, United States; ^5^Department of Preventive Medicine, Icahn School of Medicine at Mount Sinai, Friedman Brain Institute, New York, NY, United States; ^6^Department of Preventive Medicine, Icahn School of Medicine at Mount Sinai, Mindich Institute of Child Development, New York, NY, United States; ^7^The Seaver Autism Center for Research and Treatment at Mount Sinai, New York, NY, United States; ^8^Section of Epidemiology and Biostatistics, School of Population Health, The University of Auckland, Auckland, New Zealand; ^9^Faculty of Health Sciences, Department of Community Medicine, University of Tromsø, The Arctic University of Norway, Tromsø, Norway; ^10^Department of Research, Cancer Registry of Norway, Oslo, Norway; ^11^Genetic Epidemiology Group, Folkhälsan Research Center, Helsinki, Finland

**Keywords:** community assessment of psychic experiences, sunlight, UV, psychosis, epidemiology

## Abstract

**Objective:**

Sun exposure is considered the single most important source of vitamin D. Vitamin D deficiency has been suggested to play a role in the etiology of psychotic disorders. The aim of the present study was to evaluate the association between sun exposure and psychotic experiences (PEs) in a general population sample of Swedish women.

**Methods:**

The study population included participants from The Swedish Women’s Lifestyle and Health cohort study. The 20-item community assessment of psychic experiences (CAPEs) was administered between ages 30 and 50 to establish PEs. Sun exposure as measured by (1) sunbathing holidays and (2) history of sunburn was measured between ages 10 and 39. The association between sun exposure and PEs was evaluated by quantile regression models.

**Results:**

34,297 women were included in the analysis. Women who reported no sunbathing holidays and 2 or more weeks of sunbathing holidays scored higher on the CAPE scale than women exposed to 1 week of sunbathing holidays across the entire distribution, when adjusting for age and education. Similarly, compared with women who reported a history of one sunburn, the women with none or two or more sunburns showed higher scores on the CAPE scale.

**Conclusion:**

The results of the present study suggest that, in a population-based cohort of middle aged women, both low and high sun exposure is associated with increased level of positive PEs.

## Introduction

Schizophrenia and other psychotic disorders are lifelong neuropsychiatric conditions that affect 2–3% of the population (1, 2). The main characteristics of psychosis are hallucinations (false or distorted sensory experience that appear to be real), delusions (false beliefs), and changes in the form and content of thoughts and speech. Symptoms typically begin in late adolescence or early adulthood. Schizophrenia has frequently been associated with abnormalities in the dopaminergic system (3).

Vitamin D is an essential nutrient that plays an important role in many biochemical functions that contribute to aging, including bone renewal, cell proliferation, hormone balance, and cardiovascular and glucose metabolism (4). Of particular interest is accumulating evidence for the role of vitamin D in the brain (5, 6). Animal studies have shown that deficiency of prenatal vitamin D is associated with persistent changes in adult brain structure, neurochemistry, and behavior (5, 7). Evidence now links low vitamin D levels with reduced cognitive performance (8), increased risk for cancer, diabetes, cardiovascular disease, and premature death (9, 10).

The main source of vitamin D for humans is from its dermal synthesis by exposure to sunlight (11, 12), which provides up to 90% in the form of vitamin D3 (cholecalciferol) with the remainder coming from food in the form of vitamin D3 or vitamin D2 (ergocalciferol) (6, 9, 13, 14).

In order to convert 7-dehydrocholesterol to pre-vitamin D3, the skin is penetrated by solar ultraviolet B radiation (wavelength between 290 and 315 nm), which then is isomerized by heat to vitamin D3 before being carried, bound to vitamin D-binding protein, to the liver where it is converted into the main metabolite 25-hydroxyvitamin D3. Intoxication by production of excess amounts of vitamin D3 is not caused by prolonged sun exposure. Too much UV radiation from the sun does not produce excess amounts of vitamin D3 (15). Moreover, it has been argued that prolonged exposure can break down vitamin D, reducing health benefits and increasing risk of skin cancer (13). Therefore, it has been suggested that moderate sun exposure is equally effective as high sun exposure for previtamin D production (1, 5).

Murri et al. reviewed seven studies on vitamin D and psychosis and concluded that individuals with psychosis showed lower vitamin D levels than healthy controls (16), and a comprehensive meta-analysis of observation studies suggested a strong relationship between vitamin D deficiency and schizophrenia (17) Vitamin D has been found to modulate dopamine neurotransmission (5), and several studies reported an association between early life vitamin D deficiency and later psychotic disorder. Vitamin D deficiency around birth has been associated with increased risk for later schizophrenia in a Danish population-based study (18). McGrath and colleagues reported that “both low and high concentrations of neonatal vitamin D were associated with increased risk of schizophrenia.” Similarly, absence of supplementation with vitamin D before the age of one was found to be associated with later schizophrenia in males (19). However, other studies have failed to find a statistically significant association (20–22). While there are many studies reporting the association between vitamin D levels in the blood samples (18, 19), there is still a lack of studies on sun exposure, the main source of vitamin D in most countries. Only one study has reported on the relationship between sunlight exposure and schizophrenia incidence, but found no association. This study was limited in power and did not measure sun exposure at the individual level but instead used average sun exposure for a larger geographic area (23). Thus, the association between vitamin D, sun exposure, and psychosis remains debated. Previous research suggests that subclinical psychotic experiences (PEs) might be on a continuum with psychotic disorders. PEs are present in 5–15% of individuals from the general population (1, 24) and share risk factors with psychotic disorders, including social adversity (e.g., low socioeconomic status (SES), urbanicity) (24) and cognitive impairment (25). Imaging studies have also revealed pathophysiological overlaps between psychotic disorders and subclinical psychosis (26–28). Moreover, reporting PEs in early life is found to be related to increased risk of later psychotic disorders. PEs have also been associated with several other mental health disorders such as, anxiety, depression, substance misuse, suicide risk, and self-harm (24, 29–32).

Because PEs are common and associated with a range of adverse health outcomes (1, 24), it is important to study them. Moreover, since individuals with PEs generally do not receive antipsychotic medications, potential confounding by medication is avoided.

Given the limited research, the aim of this study is to investigate the association between adult life sun exposure (as a proxy for vitamin D status) and later PEs. Using a large, prospective, population-based, cohort study, we tested the hypothesis that sun exposure behaviors are associated with PEs.

## Materials and Methods

The study population originates from The Swedish Women’s Lifestyle and Health cohort. The cohort has been described in detail elsewhere (33). Briefly, the cohort included women aged 29–49 years residing in the Uppsala Health Care Region in Sweden during 1991 and 1992, of 96,000 randomly selected from four age groups (30–34, 35–39, 40–44, and 45–49 years) and invited to participate. A total of 49,261 women (52%) returned a completed questionnaire, which included demographic data and information on sun exposure behavior in different periods of life.

**Figure 1:**
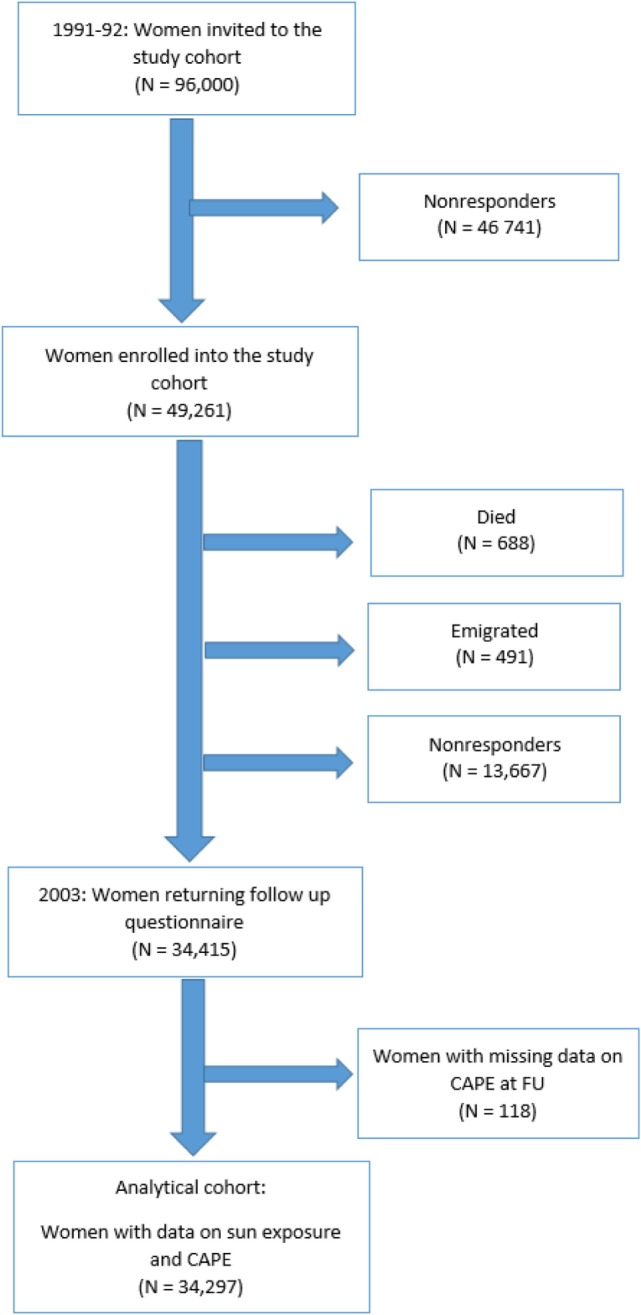
Flowchart of participants included in the Swedish Women’s Lifestyle and Health study.

In 2002/2003, a follow-up study was initiated, and women who had responded to the 1991/1992 questionnaire were alive and living in Sweden in October 2002 were contacted. Since 1991/1992, 688 women were deceased and 491 women had emigrated. 47,859 women were invited to participate. 34,415 women (72%) returned the questionnaire, which included PEs using the Community Assessment of Psychic Experiences (CAPE) scale. The Swedish Data Inspection Board and the regional Ethical Committee approved the study (Figure 1).

### Psychotic Experiences

The CAPE (http://www.cape42.homestead.com) was used to measure PEs, which is an amended version of the Peters et al.’s Delusions Inventory based on the ninth edition of the Present State Examination (34). Our version of the CAPE questionnaire contained 20 questions on psychotic-like symptoms, including only the positive symptoms. The full 42-item CAPE consists of three subscales; positive, depressive, and negative symptoms and shows satisfactory reliability in addition to construct and discriminant validity (35, 36). The 20-item positive scale strongly predicts future psychosis (37). The questions are styled in a “Do you ever feel/think” fashion. The frequency score is measured on a 4-point scale from “never (1),” “sometimes (2),” and “often (3)” to “nearly always (4).” The CAPE is a stable, valid, and reliable self-report instrument for the measurement of psychotic-like experiences in the general population based on cross-validation with interview-based data (38). The degree of distress associated with the experience was not included in the current study. The questions were translated from English into Swedish and back-translated to increase fidelity to the original scale.

The properties of the CAPE scale as applied to the current study has been examined previously (39), and the reliability was recently examined with other studies in a large meta-analysis (40). The Cronbach alpha (41) was estimated to 0.81 (0.86 standardized). Many epidemiological studies chose to group or categorize data. As a main outcome variable in this study, we calculated the mean CAPE score across all 20 items where only women with non-missing data were included.

### Sun Exposure

Sun exposure was measured at baseline by the following two variables.

#### History of Sunburn

For each 10-year period (10–19, 20–29, and 30–39 years of age), the participants were asked to report the number of times per year they had been burned by the sun (in Sweden or on beach holiday abroad) so severely that it resulted in pain or blisters that subsequently peeled by choosing from among five categories: never, one time per year, two or three times per year, four or five times per year, or six or more times per year.

#### Sunbathing Holidays

Participants reported the average number of weeks per year spent on sunbathing vacations in Sweden or in southern latitudes (typically southern Europe, e.g., Spain or Greece) for each 10-year age period by choosing from among five categories: never, 1, 2–3, 4–6, or 7 weeks/year. For each category of sun exposure history, we combined exposure across the three decades: 10–19, 20–29, and 30–39 years of age, where the last category was considered only for women aged 30 or older at cohort entry. Thus, we created three categories for each sun exposure variable: sunbathing holidays (never; 1 week/year; ≥2 weeks/year) and history of sunburn (none; 1 time; ≥2 times). This choice of categorization follows earlier studies from this cohort (42, 43).

### Confounders

Lifestyle factors controlling access to sunbathing vacation and awareness of sun-protection as well as psychosis risk can be assumed depending on SES and age. For this reason, we considered years spent in education and attained age (in years) as possible confounders in all our analyses. SES can be measured as univariate measure of educational attainment. It has been suggested that for those older than 25 years, which is the case in our cohort, educational attainment is a good proxy measure of SES (44). Evidence suggests that educational attainment after age 25 is rather constant, which means that individuals pursuing higher degrees after age 25 are usually considered as high achievers. Additionally, educational attainment is rather easy to measure and questions related to educational level are answered with higher frequency and honesty compared to income related questions. For the purpose of this study and based on available data, we measured educational attainment by years spent in education.

### Statistical Analysis

We calculated summary statistics to compare women with different levels of sun exposure. We summarized the CAPE scale by plotting side-by-side bar segmented bar charts. We included 1 bar for each of the 20 items of the CAPE scale. Each bar is further sub-divided in segments, one segment for each level of the item, where the size of each segment in the figure is proportional to the number of women with this response.

To test for an association, and estimate the change in the CAPE scale between different levels of sun-exposure (high and none vs intermediate exposure), we fitted quantile regression (45). Whereas a *t*-test or a linear regression can test for a change in the mean between different groups of exposures, quantile regression allows a comparison at points across the entire distribution, e.g., if the 75th or 90th percentile differ, not only a change in the mean. Similarly to estimating a change in mean CAPE, we estimated the change in median CAPE. More specifically, we estimated changes at the 10th, 20th, … 90th CAPE percentiles. (As a comment, 10 percent of the data will be found below the 10th percentile, 20 percent below the 20th percentile, etc., and the 50th percentile is usually referred to as the median.) We plotted the difference between exposure groups at each 10th CAPE percentile allowing a visual comparison between women subject to different levels of sun exposure. We fitted quantile regression for each of the two sun-exposure covariates, sunbathing holidays, and history of sunburns, separately. All models also included the potential confounders, years in education, and age at cohort entry.

There are several advantages to using quantile regression when comparing two exposure groups. First, using quantile regression allows us to compare not only the mean but the entire CAPE distribution. We hypothesized that a change in sun exposure would primarily be seen in the right side of the CAPE distribution, which is where the women with most PEs are to be found. Second, quantile regression is a technique robust against single gross outliers and do not rely on assumptions of the data following a specific data distribution such as the Gaussian or the binomial.

For both sun exposure variables, the intermediate and most common sun exposure (1 week or 1 time, respectively) were chosen as the reference category. For each sun-exposure variable and each 10th percentile comparing two levels of sun exposure (high vs intermediate and none vs intermediate), we calculated the difference in 10th percentiles together with the two-sided 95% confidence intervals using bootstrap (46).

We also performed a set of sensitivity analyses. First, we explored the exposure in different ages of life by refitting the models described above, but for sun exposure during ages 10–19 (adolescence period) and ages 20–29, separately (adulthood period). Second, of the 49,259 women in the cohort 1991/92, 34,402 (70%) women returned for the follow-up in 2004 when PE was measured. Missing values and dropouts due to a mechanism acting as a confounder between sun exposure and PE and a not missing at random could potentially bias the results (47). We did not find any logical reason why this should be the case. Still, in a sensitivity analysis, we addressed this potential problem by first comparing the covariate distribution among the women entering the cohort 1991 with the women returning for the follow-up. Inverse probability weighting is one of several methods that can reduce this bias (48). In this method, complete cases are weighted by the inverse of their probability of being a complete case. We applied this method. Statistical analyses were performed using the SAS version 9.4. All tests of statistical hypotheses were done on the two-sided 5% level of significance.

## Results

The population consisted of 34,297 women who reported data on the CAPE questionnaire at follow-up stage of the study in 2003. There were 1,252 women with missing values on sun exposure and on the confounder (years in education). When filling in the follow-up questionnaire, the mean age of women was 40.4, SD = 5.7. The average number of years in education was 12.4 years SD = 3.0 (Table 1).

**Table 1:** Cohort characteristics by levels of sun exposure at ages 10–39 years.

Sun exposure variable	Cohort, number of women	Age mean (SD)	Education^a^ mean (SD)
Annual cumulative number of weeks spent on sunbathing holidays	34,297		
Never	3,913	41.7 (5.7)	11.7 (3.3)
1 week	10,126	40.5 (5.6)	12.4 (3.0)
≥2 weeks	19,706	40 (5.7)	12.6 (2.9)
History of sunburns	34,297		
None	2,683	41.8 (5.8)	11.3 (2.9)
1 time	18,722	40.7 (5.7)	12.4 (3.0)
≥2 times	12,192	39.5 (5.7)	12.7 (2.9)

*Missing values for sunbathing holidays N = 552*.

*Missing values for sunburns N = 16,261*.

*N, number of women*.

*^a^Average years spent in education*.

*SD, Standard deviation*.

### Distribution of CAPE Scores among the Swedish Women

**Figure 2:**
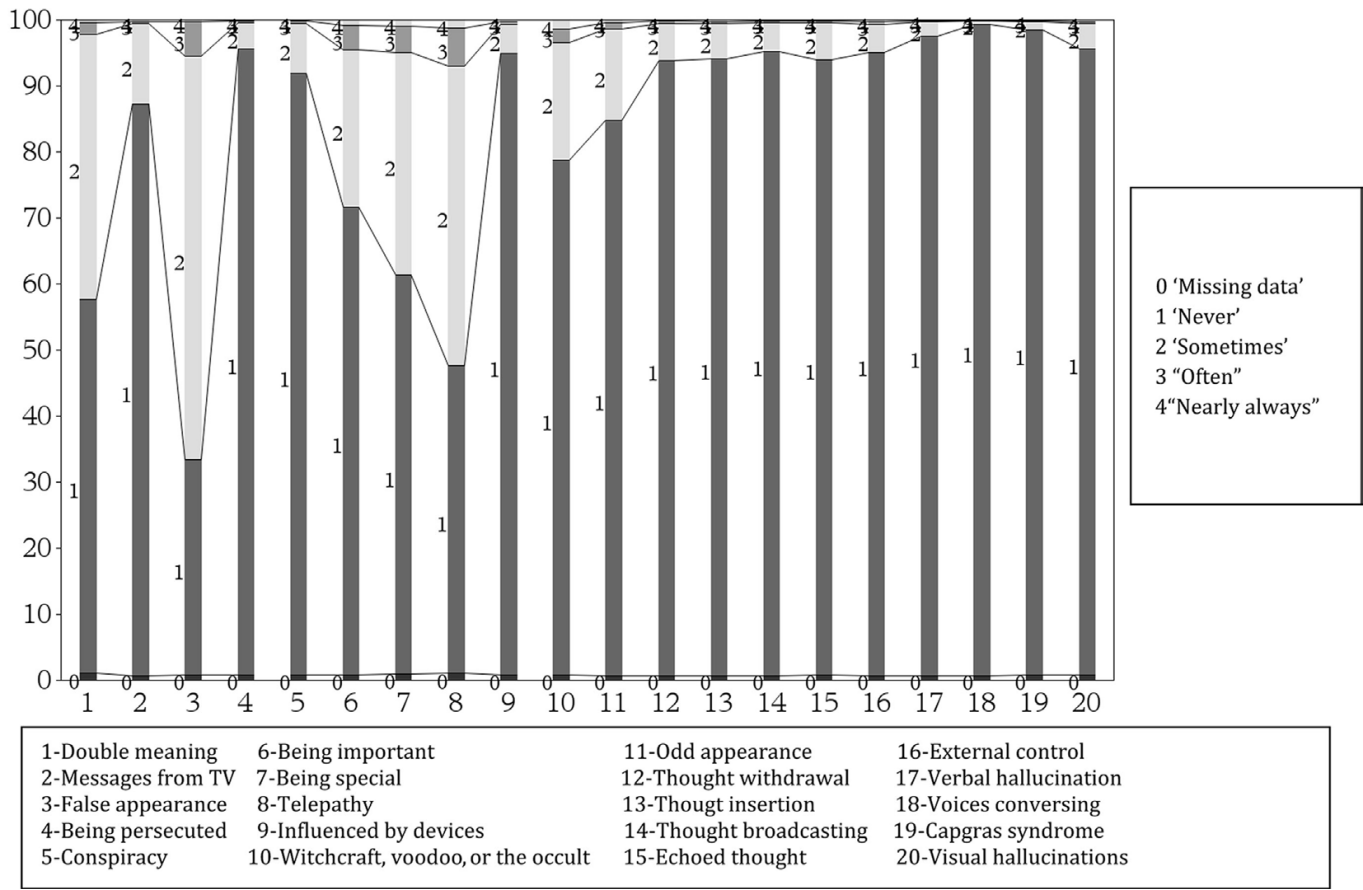
Response frequencies (%) of community assessment of psychic experiences (20 items) scores among 34,297 population-based cohort of Swedish middle aged women.

The distribution of the 20 items defining the PEs is presented in Figure 2. The mean response scores for each item of the CAPE scale is presented in Table S2 in Supplementary Material. In the entire population, the distribution of CAPE scores was mildly skewed; the mean (SD) for all subjects was 1.19 (0.19). In an ideal population entirely free of psychotic symptoms, each of the 20 items in the CAPE scale should have the weight 1:20. Items related to “Messages from TV,” “Witchcraft, voodoo, or the occult,” and “Odd appearance” had average of 1.1–1.3; “Being important,” “Being special,” and “Double meaning” an average of 1.3 and 1.5 while items “Telepathy” and “False appearance” had an population average of 1.6 and 1.7 (Figure 2; Table S1 in Supplementary Material).

### History of Sunburns and CAPE Scores

**Figure 3:**
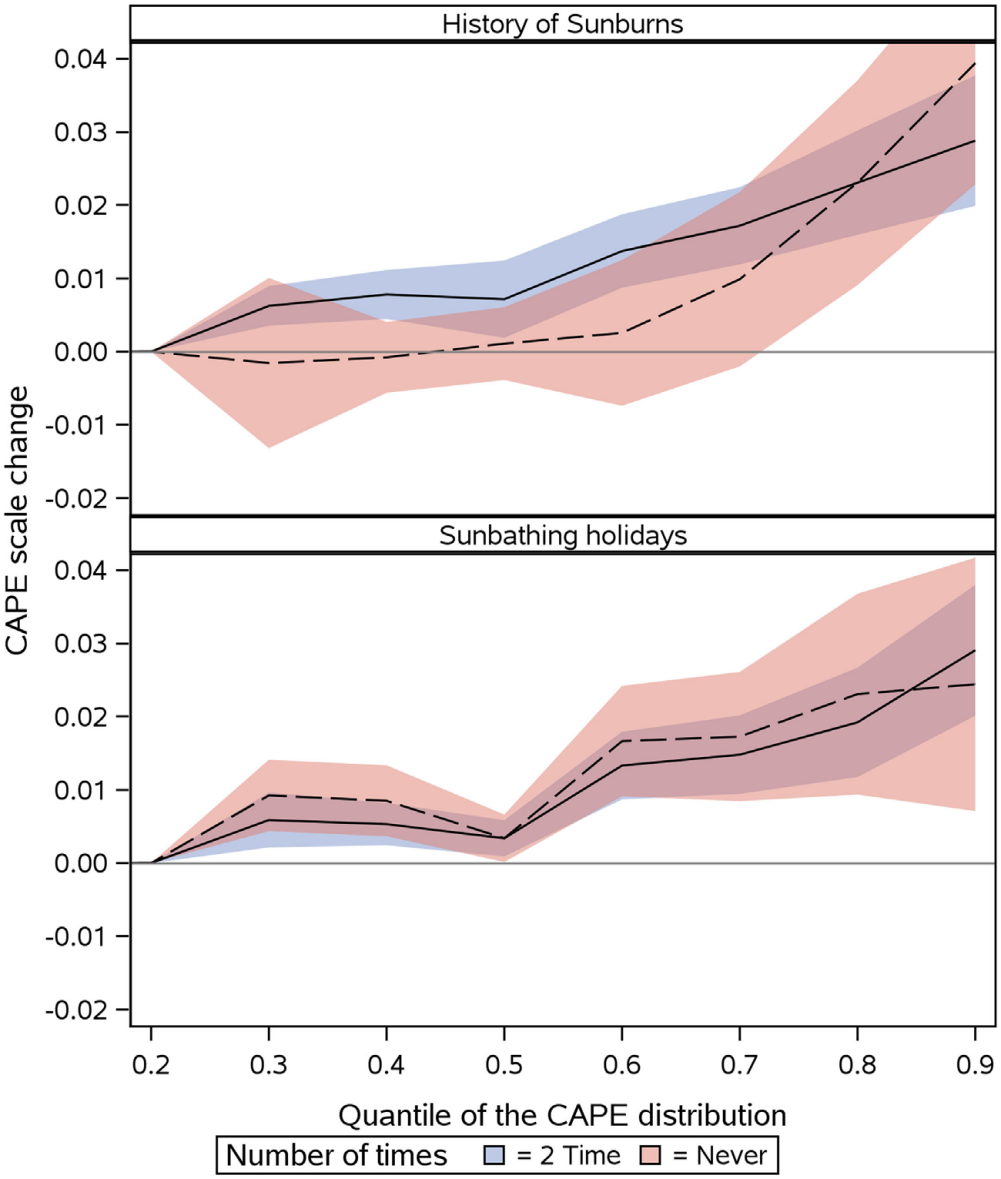
Change in CAPE Scale (*y*-axis) at different quantiles (*x*-axis) of the CAPE distribution for two measures of sun exposures at age 10–39. (1) Top panel: history of Sunburns, more than once (blue), or none (red) compared to once, and, (2) bottom panel: sunbathing holidays >1 week/year (blue) or never (red) compared to 1 week/year. Notes: quantile 0.5 is the median of the CAPE distribution. 90% of the CAPE distributions lower than 0.9. CAPE, community assessment of psychic experiences.

Compared with women who reported a history of one sunburn, the women with “None” or ≥2 sunburn showed higher scores on the CAPE scale with more women in the right part of the distribution, which is where the women with the most PEs are found (Figure 3, top panel). The PEs median (50th percentile), 80th percentile, and 90th percentile difference for ≥2 sunburn vs 1 sunburn was estimated to 0.01 (95% CI: 0.002–0.012), 0.02 (95% CI: 0.016–0.030), and 0.03 (95% CI: 0.019–0.037), respectively. The PEs for women reporting one sunburn vs none were higher and statistically significant at 80th and 90th percentile only and was estimated to 0.02 (95% CI: 0.009–0.037), 0.04 (95% CI: 0.022–0.056), respectively. Results for all 10th percentiles are presented in Table S2 in Supplementary Material.

### History of Sunbathing Holidays and CAPE Scores

Similarly, compared with women exposed to 1 week of sunbathing holidays, the women with none or ≥2 weeks showed higher scores on CAPE scale (Figure 3, bottom panel). The results were statistically significant across the entire distribution of the CAPE scale. The PEs median (50th percentile), 80th percentile, and 90th percentile difference for ≥2 vs 1 week of sunbathing vacation was estimated to 0.003 (95% CI: 0.009–0.006), 0.02 (95% CI: 0.012–0.026), and 0.03 (95% CI: 0.020–0.038), respectively. The PEs median (50th percentile), 80th, and 90th percentile difference for “Never” vs “1 week” of sunbathing vacation was estimated to 0.03 (95% CI: 0.000–0.006), 0.02 (95% CI: 0.009–0.036), and 0.02 (95% CI: 0.007–0.041), respectively. Results for all 10th percentiles are presented in Table S3 in Supplementary Material.

### Sensitivity Analysis

The results were comparable when restricting the analyses to sun exposure at specific age periods; 10–19 years of age and 20–29 years of age (Figures S1 and S2 in Supplementary Material).

For the analysis using inverse probability weighting, we predicted probabilities of participating in the follow-up part of the study based on subject characteristics. After applying the weights to the quantile regression models, we concluded that the estimates did not differ from the original estimates (Figures S3 in Supplementary Material).

## Discussion

In the Nordic countries, as elsewhere, sun exposure is by far the most important source of vitamin D. In a population-based cohort of over 30,000 women, we found an association between cumulative measures of sun exposure at ages 10–39 years and positive PEs later in life. We observed a U-shaped association where women with low sun exposure as well as women with high sun exposure reported more PEs compared to women with an intermediate level of sun exposure.

The U-shaped association is consistent with previous studies on vitamin D and health outcomes (18). The non-linear association presented in this study might suggest against a possible causal link. However, the link between sun exposure and health outcomes is often reported as non-linear with a balanced exposure showing higher effects (49, 15).

An earlier study by Hedelin et al. using the same cohort of women showed that vitamin D intake from the diet was statistically significantly associated with a decreased relative risk of both medium and high levels of psychotic symptoms (50). The conflicting finding of the present study could possibly be attributed to any of the following: (1) different measures were used as proxies for vitamin D—the present study only considered the sun exposure variables (and not diet) as a main source for vitamin D levels (9, 13, 14); (2) the focus of the present study was detailed assessment of the entire distribution of the CAPE scale whereas Hedelin et al. reported results for the categorized CAPE scale (low, middle, high level). We believe that this categorization of responses could exclude important information. Nevertheless, both studies using the same cohort and the same underlying CAPE scale reported a positive association. One conclusion that can be drawn from our results is that an intermediate sun exposure could be protective for PEs. The study by Hedelin et al. showed a J-shaped association, with reduced risk for an intermediate intake of vitamin D (fish intake) (13).

Consequently, a major strength of our study is the assessment of sun exposure during different periods of life. The information on sun exposure habits was gathered at the start of the study, which minimizes the risk of reverse causality. Still, our study possibly includes non-differential (or random) measurement error since sun exposure information was collected retrospectively, which might attenuate the examined associations.

Another major strength of this study is use of an unselected, large, population-based cohort randomly drawn from the Swedish National Population Register and followed for over 10 years.

In the present study, we performed a quantile regression. This method allows for comparisons not only of mean values between two groups but also for group comparisons in the tails of a data distribution. This is often of interest for epidemiologists who may want to compare the point where the top 10% of the study participants are found rather than where the average degree of response is found. This method is important when calculating a non-linear risk factor (45).

Our study has several limitations. We had no biological data on vitamin D levels, which could have established a clear link to vitamin D, although it is well known that sun exposure is the most important determinant of vitamin D status (9, 13). Additionally, the assumption was made that sun exposure habits did not change over time and consequently, information from one assessment alone was used in the models. This is a common assumption in cohort studies and tends to underestimate risk. However, the questionnaire did take changes into consideration, as it asked about exposure in different decades.

The current study was based on multiple exposure factors (several sun exposure variables) as proxies for vitamin D level and their relationship with PEs. The estimates were adjusted for confounding effects and each exposure variable was assessed independently. Still, our results should be interpreted cautiously. Even well-designed observational studies can be influenced by residual confounding. There may also be several unknown factors that we did not take into consideration in our study, which could influence PEs and be related to sun exposure. Another limitation is that there is only a small number of published studies reporting the association between questionnaires-based sun exposure and vitamin D. For example, Glanz et al. confirmed the validity of self-reports for UV radiation and a Norwegian study by Brustad et al. found that questionnaire-based reports of sun exposure was considered a strong predictor of vitamin D (51). The same study further reported that “staying at more southern latitudes during the previous summer was also positively associated with vitamin D in the blood” (51) (sun exposure self-reports included in the present study consist of questions related to time spent on sunny holidays in Southern latitudes).

As suggested by The American Cancer Society, excessive sun exposure is considered as potential risk factor for the malignant melanoma. A previous study using the same cohort as ours linked sun exposure to malignant melanoma (43).

We did not have data on the full CAPE scale, but only the positive symptoms. Moreover, the degree of distress associated with PEs was not included in the current study, which could have allowed a more detailed analysis on items and dimensions. The CAPE scale of PEs has been reported to be correlated with general psychopathology measures, including depression. The association between the positive and the depressive dimension in CAPE, which we could not include for practical reasons, is fairly low when distress associated with positive symptoms is held constant (*r* = 0.25) (52). As a consequence, the dimension of positive symptoms is believed to be considered an independent dimension. Further, data on PEs were not measured at baseline therefore reverse causality could be a potential explanation of findings.

In terms of generalizability of the present study, males were not included and previous reports suggest a gender difference in the incidence of psychosis. However, in general population samples, PEs are found to be equally distributed among males and females (34, 53).

## Conclusion

Both low and high sun exposure is associated with increased levels of positive Psychotic experiences. Previous studies have reported associations between vitamin D and increased risk for health problems, such as schizophrenia, as well as clinical symptoms in patients with mental health problems. However, there is a lack of previous studies on how sun exposure, the main source of vitamin D, influences Psychotic experiences in the general population. This study contributes to the literature by showing, in the general population of Swedish women, that sun exposure has a U-shaped association with positive Psychotic experiences.

## Ethics Statement

This study was carried out in accordance with the recommendations of “Institutional/Ethics Review Boards at Uppsala University and Karolinska Institutet” with written informed consent from all subjects. All subjects gave written informed consent in accordance with the Declaration of Helsinki. The protocol was approved by the “Ethics Review Boards at Uppsala University and Karolinska Institutet.”

## Author Contributions

IP was responsible for the conception of the study, interpretation of the data, manuscript design, and statistical analysis. SS was responsible for the manuscript design, statistical analysis and data interpretation. AR was responsible for the manuscript drafting, data interpretation, and revision for important intellectual content. AD and RS were responsible for providing important intellectual content throughout the manuscript’s production and for approval of the final version. EW was responsible for the study design, manuscript drafting, and revision for important intellectual content and for approval of the final version.

## Conflict of Interest Statement

The authors declare that the research was conducted in the absence of any commercial or financial relationships that could be construed as a potential conflict of interest.

## References

[1] McGrath JJ Saha S Al-Hamzawi A Alonso J Bromet EJ Bruffaerts R Psychotic experiences in the general population: a cross-national analysis based on 31 261 respondents from 18 countries. JAMA Psychiatry (2015) 72:697–705. 10.1001/jamapsychiatry.2015.0575 26018466 PMC5120396

[2] Perälä J Suvisaari J Saarni SI Kuoppasalmi K Isometsä E Pirkola S Lifetime prevalence of psychotic and bipolar I disorders in a general population. Arch Gen Psychiatry (2007) 64:19–28. 10.1001/archpsyc.64.1.19 17199051

[3] van Os J Kapur S. Schizophrenia. Lancet (2009) 374:635–45. 10.1016/S0140-6736(09)60995-8 19700006

[4] Berridge MJ. Vitamin D cell signalling in health and disease. Biochem Biophys Res Commun (2015) 460:53–71. 10.1016/j.bbrc.2015.01.008 25998734

[5] Eyles D Brown J Mackay-Sim A McGrath J Feron F Vitamin d3 and brain development. Neuroscience (2003) 118:641–53. 10.1016/S0306-4522(03)00040-X 12710973

[6] Holick MF Chen TC. Vitamin D deficiency: a worldwide problem with health consequences. Am J Clin Nutr (2008) 87:1080S–6S. 18400738 10.1093/ajcn/87.4.1080S

[7] Burne THJ Becker A Brown J Eyles DW Mackay-Sim A McGrath JJ. Transient prenatal Vitamin D deficiency is associated with hyperlocomotion in adult rats. Behav Brain Res (2004) 154:549–55. 10.1016/j.bbr.2004.03.023 15313044

[8] Annweiler C Schott AM Allali G Bridenbaugh SA Kressig RW Allain P Association of vitamin D deficiency with cognitive impairment in older women: cross-sectional study. Neurology (2010) 74:27–32. 10.1212/WNL.0b013e3181beecd3 19794127

[9] LeBlanc E Chou R Zakher B Daeges M Pappas M Screening for Vitamin D Deficiency. (2014). Available from: http://www.ncbi.nlm.nih.gov/books/NBK263419/25521000

[10] Pludowski P Holick MF Pilz S Wagner CL Hollis BW Grant WB Vitamin D effects on musculoskeletal health, immunity, autoimmunity, cardiovascular disease, cancer, fertility, pregnancy, dementia and mortality-a review of recent evidence. Autoimmun Rev (2013) 12:976–89. 10.1016/j.autrev.2013.02.004 23542507

[11] Touvier M Deschasaux M Montourcy M Sutton A Charnaux N Kesse-Guyot E Determinants of vitamin D status in Caucasian adults: influence of sun exposure, dietary intake, sociodemographic, lifestyle, anthropometric, and genetic factors. J Invest Dermatol (2015) 135:378–88. 10.1038/jid.2014.400 25211176

[12] Lips P van Schoor NM de Jongh RT. Diet, sun, and lifestyle as determinants of vitamin D status. Ann N Y Acad Sci (2014) 1317:92–8. 10.1111/nyas.12443 24814938

[13] Holick MF. Sunlight and vitamin D for bone health and prevention of autoimmune diseases, cancers, and cardiovascular disease. Am J Clin Nutr (2004) 80:1678S–88S. 15585788 10.1093/ajcn/80.6.1678S

[14] Umhau JC George DT Heaney RP Lewis MD Ursano RJ Heilig M Low vitamin D status and suicide: a case-control study of active duty military service members. PLoS One (2013) 8:e51543. 10.1371/journal.pone.0051543 23308099 PMC3537724

[15] Sivamani RK Crane LA Dellavalle RP. The benefits and risks of ultraviolet tanning and its alternatives: the role of prudent sun exposure. Dermatol Clin (2009) 27:149–54, vi. 10.1016/j.det.2008.11.008 19254658 PMC2692214

[16] Belvederi Murri M Respino M Masotti M Innamorati M Mondelli V Pariante C Vitamin D and psychosis: mini meta-analysis. Schizophr Res (2013) 150:235–9. 10.1016/j.schres.2013.07.017 23906618

[17] Valipour G Saneei P Esmaillzadeh A. Serum vitamin D levels in relation to schizophrenia: a systematic review and meta-analysis of observational studies. J Clin Endocrinol Metab (2014) 99:3863–72. 10.1210/jc.2014-1887 25050991

[18] McGrath JJ Eyles DW Pedersen CB Anderson C Ko P Burne TH Neonatal vitamin D status and risk of schizophrenia: a population-based case-control study. Arch Gen Psychiatry (2010) 67:889–94. 10.1001/archgenpsychiatry.2010.110 20819982

[19] McGrath J Saari K Hakko H Jokelainen J Jones P Järvelin M-R Vitamin D supplementation during the first year of life and risk of schizophrenia: a Finnish birth cohort study. Schizophr Res (2004) 67:237–45. 10.1016/j.schres.2003.08.005 14984883

[20] Norelli LJ Coates AD Kovasznay BM A comparison of 25-hydroxyvitamin D serum levels in acute and long-stay psychiatric inpatients: a preliminary investigation. E Spen Eur E J Clin Nutr Metab (2010) 5:e187–9. 10.1016/j.eclnm.2010.04.003

[21] Ryan AS Astwood JD Gautier S Kuratko CN Nelson EB Salem N Effects of long-chain polyunsaturated fatty acid supplementation on neurodevelopment in childhood: a review of human studies. Prostaglandins Leukot Essent Fatty Acids (2010) 82:305–14. 10.1016/j.plefa.2010.02.007 20188533

[22] Schneider B Weber B Frensch A Stein J Fritze J. Vitamin D in schizophrenia, major depression and alcoholism. J Neural Transm (2000) 107:839–42. 10.1007/s007020070063 11005548

[23] Kendell R Exposure to sunlight, vitamin D and schizophrenia. Schizophr Res (2002) 54:193–8. 10.1016/S0920-9964(01)00264-X 11950543

[24] van Os J Linscott RJ Myin-Germeys I Delespaul P Krabbendam L. A systematic review and meta-analysis of the psychosis continuum: evidence for a psychosis proneness-persistence-impairment model of psychotic disorder. Psychol Med (2009) 39:179–95. 10.1017/S0033291708003814 18606047

[25] Simons CJP Jacobs N Jolles J van Os J Krabbendam L. Subclinical psychotic experiences and cognitive functioning as a bivariate phenotype for genetic studies in the general population. Schizophr Res (2007) 92:24–31. 10.1016/j.schres.2007.01.008 17346933

[26] DeRosse P Karlsgodt KH Examining the psychosis continuum. Curr Behav Neurosci Rep (2015) 2:80–9. 10.1007/s40473-015-0040-7 26052479 PMC4454466

[27] Mittal VA Orr JM Turner JA Pelletier AL Dean DJ Lunsford-Avery J Striatal abnormalities and spontaneous dyskinesias in non-clinical psychosis. Schizophr Res (2013) 151:141–7. 10.1016/j.schres.2013.10.003 24156901 PMC3855894

[28] Jacobson S Kelleher I Harley M Murtagh A Clarke M Blanchard M Structural and functional brain correlates of subclinical psychotic symptoms in 11–13 year old schoolchildren. Neuroimage (2010) 49:1875–85. 10.1016/j.neuroimage.2009.09.015 19770054

[29] Rössler W Hengartner MP Ajdacic-Gross V Haker H Gamma A Angst J. Sub-clinical psychosis symptoms in young adults are risk factors for subsequent common mental disorders. Schizophr Res (2011) 131:18–23. 10.1016/j.schres.2011.06.019 21757323

[30] Olfson M Lewis-Fernández R Weissman MM Feder A Gameroff MJ Pilowsky D Psychotic symptoms in an urban general medicine practice. Am J Psychiatry (2002) 159:1412–9. 10.1176/appi.ajp.159.8.1412 12153836

[31] Saha S Chant D Welham J McGrath J. A systematic review of the prevalence of schizophrenia. PLoS Med (2005) 2:e141. 10.1371/journal.pmed.0020141 15916472 PMC1140952

[32] Saha S Scott JG Johnston AK Slade TN Varghese D Carter GL The association between delusional-like experiences and suicidal thoughts and behaviour. Schizophr Res (2011) 132:197–202. 10.1016/j.schres.2011.07.012 21813264

[33] Roswall N Sandin S Adami H-O Weiderpass E Cohort profile: the Swedish women’s lifestyle and health cohort. Int J Epidemiol (2015):dyv089 10.1093/ije/dyv089 26066328

[34] Peters ER Joseph SA Garety PA Measurement of delusional ideation in the normal population: introducing the PDI (Peters et al. Delusions Inventory). Schizophr Bull (1999) 25:553–76. 10.1093/oxfordjournals.schbul.a033401 10478789

[35] Brenner K Schmitz N Pawliuk N Fathalli F Joober R Ciampi A Validation of the English and French versions of the community assessment of psychic experiences (CAPE) with a Montreal community sample. Schizophr Res (2007) 95:86–95. 10.1016/j.schres.2007.06.017 17693059

[36] Fonseca-Pedrero E Paino M Lemos-Giráldez S Muñiz J. Validation of the community assessment psychic experiences-42 (CAPE-42) in Spanish college students and patients with psychosis. Actas Esp Psiquiatr (2012) 40:169–76. 22851477

[37] Capra C Kavanagh DJ Hides L Scott J. Brief screening for psychosis-like experiences. Schizophr Res (2013) 149:104–7. 10.1016/j.schres.2013.05.020 23830544

[38] Konings M Bak M Hanssen M van Os J Krabbendam L. Validity and reliability of the CAPE: a self-report instrument for the measurement of psychotic experiences in the general population. Acta Psychiatr Scand (2006) 114:55–61. 10.1111/j.1600-0447.2005.00741.x 16774662

[39] Therman S Suvisaari J Hultman CM. Dimensions of psychotic experiences among women in the general population. Int J Methods Psychiatr Res (2014) 23:62–8. 10.1002/mpr.1427 24375586 PMC6878595

[40] Mark W Toulopoulou T Psychometric properties of “community assessment of psychic experiences”: review and meta-analyses. Schizophr Bull (2016) 42:34–44. 10.1093/schbul/sbv088 26150674 PMC4681550

[41] Cronbach LJ Coefficient alpha and the internal structure of tests. Psychometrika (1951) 16:297–334. 10.1007/BF02310555

[42] Yang L Lof M Veierød MB Sandin S Adami H-O Weiderpass E. Ultraviolet exposure and mortality among women in Sweden. Cancer Epidemiol Biomarkers Prev (2011) 20:683–90. 10.1158/1055-9965.EPI-10-0982 21297041

[43] Yang L Veierød MB Löf M Sandin S Adami H-O Weiderpass E. Prospective study of UV exposure and cancer incidence among Swedish women. Cancer Epidemiol Biomarkers Prev (2011) 20:1358–67. 10.1158/1055-9965.EPI-11-0071 21551241

[44] Measuring Socioeconomic Status. (2017). Available from: http://www.esourceresearch.org/Portals/0/Uploads/Documents/Public/Oakes_FullChapter.pdf

[45] Cade BS Noon BR A gentle introduction to quantile regression for ecologists. Front Ecol Environ (2003) 1:412–20. 10.1890/1540-9295(2003)001[0412:AGITQR]2.0.CO;2

[46] He X Hu F Markov chain marginal bootstrap. J Am Stat Assoc (2002) 97:783–95. 10.1198/016214502388618591

[47] Little RJA Rubin DB Statistical Analysis with Missing Data. (1987). Available at: http://www.gbv.de/dms/ilmenau/toc/33682193X.PDF

[48] Seaman SR White IR. Review of inverse probability weighting for dealing with missing data. Stat Methods Med Res (2013) 22(3):278–95. 10.1177/0962280210395740 21220355

[49] WHO. The Known Health Effects of UV. (2015). Available from: http://www.who.int/uv/faq/uvhealtfac/en/

[50] Hedelin M Löf M Olsson M Lewander T Nilsson B Hultman CM Dietary intake of fish, omega-3, omega-6 polyunsaturated fatty acids and vitamin D and the prevalence of psychotic-like symptoms in a cohort of 33,000 women from the general population. BMC Psychiatry (2010) 10:38. 10.1186/1471-244X-10-38 20504323 PMC2889879

[51] Brustad M Alsaker E Engelsen O Aksnes L Lund E Vitamin D status of middle-aged women at 65-71°N in relation to dietary intake and exposure to ultraviolet radiation. Public Health Nutr (2004) 7:327–35. 10.1079/PHN2003536 15003141

[52] Stefanis NC Hanssen M Smirnis NK Avramopoulos DA Evdokimidis IK Stefanis CN Evidence that three dimensions of psychosis have a distribution in the general population. Psychol Med (2002) 32:347–58. 10.1017/S0033291701005141 11866327

[53] Varghese D Scott J McGrath J. Correlates of delusion-like experiences in a non-psychotic community sample. Aust N Z J Psychiatry (2008) 42:505–8. 10.1080/00048670802050595 18465377

