# Supplementary material for: Sun Exposure and Psychotic Experiences
Source: Front Psychiatry. 2017 Jun 19;8:107. doi: 10.3389/fpsyt.2017.00107 (PMC5474873; doi:10.3389/fpsyt.2017.00107)
Supplement: Supplementary file 1 [file Data_Sheet_1.docx]

# Supplementary material

eTable1 Mean values for 20 items of the CAPE scale.

| CAPE items | Label | N | Mean | Std Dev |
| --- | --- | --- | --- | --- |
| 3 | False appearance | 34118 | 1.7 | 0.6 |
| 8 | Telepathy | 34050 | 1.6 | 0.7 |
| 1 | Double meaning | 34025 | 1.5 | 0.6 |
| 7 | ’Being special’ | 34111 | 1.5 | 0.6 |
| 6 | Being important | 34136 | 1.3 | 0.6 |
| 10 | Witchcraft, voodoo or the occult | 34131 | 1.3 | 0.6 |
| 11 | Odd appearance | 34182 | 1.2 | 0.4 |
| 2 | Messages from TV | 34175 | 1.1 | 0.4 |
| 4 | Being persecuted | 34152 | 1.1 | 0.3 |
| 5 | Conspiracy | 34137 | 1.1 | 0.3 |
| 9 | Influenced by devices | 34147 | 1.1 | 0.3 |
| 12 | Thought withdrawal | 34178 | 1.1 | 0.3 |
| 13 | Thought insertion | 34183 | 1.1 | 0.3 |
| 14 | Thought broadcasting | 34186 | 1.1 | 0.3 |
| 15 | Echoed thought | 34153 | 1.1 | 0.3 |
| 16 | External control | 34162 | 1.1 | 0.3 |
| 20 | Visual hallucinations | 34131 | 1.1 | 0.3 |
| 17 | Verbal hallucinations | 34168 | 1 | 0.2 |
| 18 | Voices conversing | 34163 | 1 | 0.2 |
| 19 | Capgras syndrome | 34154 | 1 | 0.2 |

eTable 2 Quantile regression estimates for PLEs by levels of history of sunburns (adjusted for age and education).

| **Quantile** | **Categories of history of sunburns (‘1 time’ as a comparison group)** | **Estimate** | **95% Lower Confidence Limit** | **95% Upper Confidence Limit** | **P value** |
| --- | --- | --- | --- | --- | --- |
| 0.1 | None | 0 | 0 | 0 | 0.0006 |
| 0.1 | ≥2 times | 0 | 0 | 0 | 0.0008 |
| 0.2 | None | 0 | 0 | 0 | 0.0002 |
| 0.2 | ≥2 times | 0 | 0 | 0 | 0.0012 |
| 0.3 | None | -0.0016 | -0.0132 | 0.0101 | 0.7923 |
| 0.3 | ≥2 times | 0.0063 | 0.0035 | 0.009 | <.0001 |
| 0.4 | None | -0.0008 | -0.0056 | 0.0041 | 0.7522 |
| 0.4 | ≥2 times | 0.0078 | 0.0045 | 0.0112 | <.0001 |
| 0.5 | None | 0.0011 | -0.0039 | 0.006 | 0.6643 |
| 0.5 | ≥2 times | 0.0072 | 0.0019 | 0.0124 | 0.0079 |
| 0.6 | None | 0.0026 | -0.0074 | 0.0125 | 0.6147 |
| 0.6 | ≥2 times | 0.0137 | 0.0087 | 0.0187 | <.0001 |
| 0.7 | None | 0.0099 | -0.002 | 0.0218 | 0.1035 |
| 0.7 | ≥2 times | 0.0172 | 0.0119 | 0.0225 | <.0001 |
| 0.8 | None | 0.0231 | 0.0091 | 0.0371 | 0.0012 |
| 0.8 | ≥2 times | 0.0231 | 0.0159 | 0.0302 | <.0001 |
| 0.9 | None | 0.0394 | 0.0228 | 0.056 | <.0001 |
| 0.9 | ≥2 times | 0.0288 | 0.0199 | 0.0377 | <.0001 |

eTable 3 Quantile regression estimates for PEs by levels of sunbathing holidays (adjusted for age and education).

| **Quantile** | **Categories of sun exposure (‘1 week’ as a comparison group)** | **Estimate** | **95% Lower Confidence Limit** | **95% Upper Confidence Limit** | **P value** |
| --- | --- | --- | --- | --- | --- |
| 0.1 | Never | 0 | 0 | 0 | 0.001 |
| 0.1 | ≥2 weeks | 0 | 0 | 0 | 0.0035 |
| 0.2 | Never | 0 | 0 | 0 | 0.0012 |
| 0.2 | ≥2 weeks | 0 | 0 | 0 | 0.0044 |
| 0.3 | Never | 0.0092 | 0.0044 | 0.0141 | 0.0002 |
| 0.3 | ≥2 weeks | 0.0059 | 0.0021 | 0.0096 | 0.002 |
| 0.4 | Never | 0.0085 | 0.0037 | 0.0134 | 0.0006 |
| 0.4 | ≥2 weeks | 0.0053 | 0.0024 | 0.0082 | 0.0003 |
| 0.5 | Never | 0.0034 | 0.0001 | 0.0067 | 0.0428 |
| 0.5 | ≥2 weeks | 0.0034 | 0.0009 | 0.0059 | 0.0078 |
| 0.6 | Never | 0.0167 | 0.0091 | 0.0242 | <.0001 |
| 0.6 | ≥2 weeks | 0.0133 | 0.0087 | 0.018 | <.0001 |
| 0.7 | Never | 0.0173 | 0.0084 | 0.0261 | 0.0001 |
| 0.7 | ≥2 weeks | 0.0148 | 0.0094 | 0.0202 | <.0001 |
| 0.8 | Never | 0.0231 | 0.0094 | 0.0368 | 0.001 |
| 0.8 | ≥2 weeks | 0.0192 | 0.0118 | 0.0267 | <.0001 |
| 0.9 | Never | 0.0244 | 0.0071 | 0.0417 | 0.0056 |
| 0.9 | ≥2 weeks | 0.0291 | 0.0202 | 0.038 | <.0001 |

**eFigure 1 Distribution of PEs by levels of sun exposure at age 10-19 (history of sunburns at age 10-19 compared to ‘1 time’ and sunbathing holidays at age 10-19 compared to holidays ‘1 week’ (adjusted for age and education).**


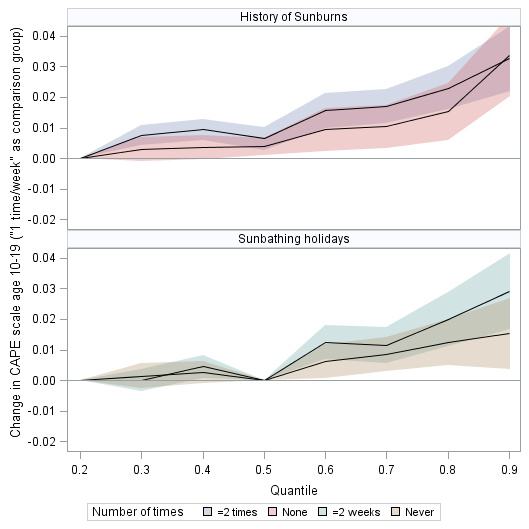


eFigure 2 Distribution of PEs by sun exposure at age 20-39 (history of sunburns at age 20-39 compared to ‘1 time’ and sunbathing holidays at age 20-39 compared to holidays ‘1 week’ (adjusted for age and education).


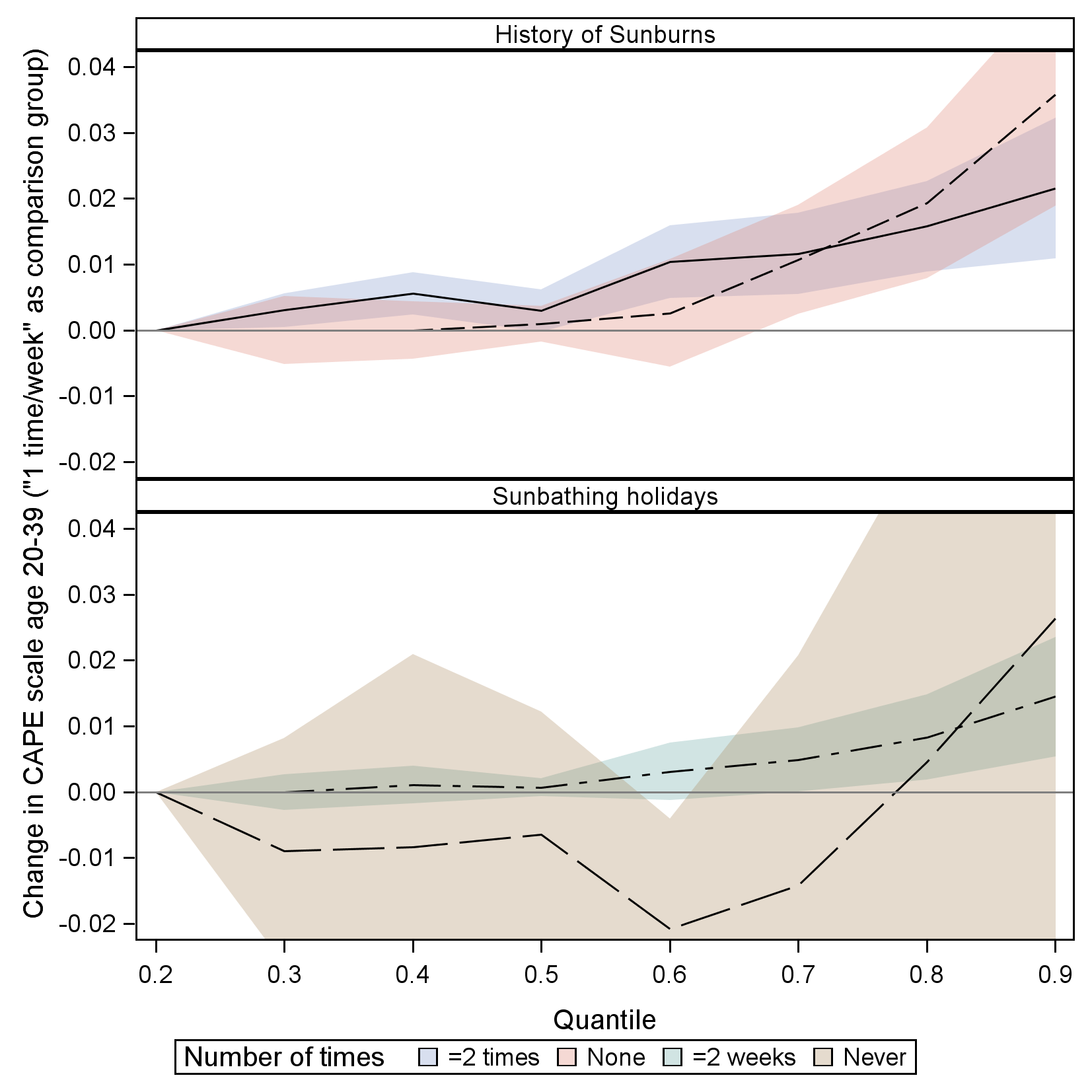


**eFigure 3 Distribution of PEs by levels of sun exposure at age 20-39 (history of sunburns at age 20-39 compared to ‘1 time’ and sunbathing holidays at age 20-39 compared to holidays ‘1 week’ (after applying ‘Inverse Probability Weighting’ method).**


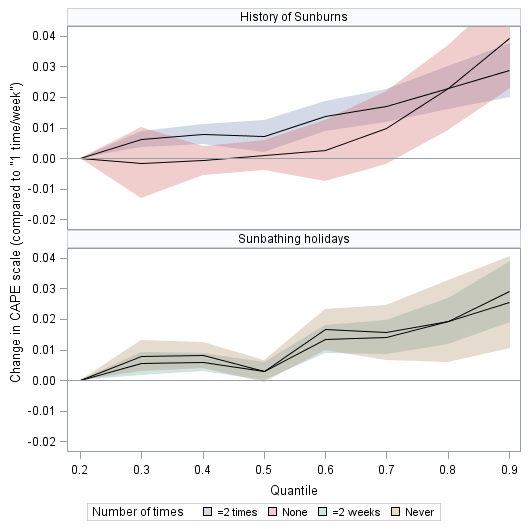


*Notes: Probability weights were calculated using logistic regression and including covariates: age, education and body mass index.*
